# Gene Therapy for Cystic Fibrosis: Recent Advances and Future Prospects

**DOI:** 10.32607/actanaturae.11708

**Published:** 2023

**Authors:** M. A. Lomunova, P. M. Gershovich

**Affiliations:** JSC BIOCAD, Saint-Petersburg, 198515 Russian Federation

**Keywords:** gene therapy, cystic fibrosis, CFTR, viral vector, nanoparticles

## Abstract

Gene replacement therapies are novel therapeutic approaches that seek to tackle hereditary diseases caused by a congenital deficiency in a particular gene, when a functional copy of a gene can be delivered to the cells and tissues using various delivery systems. To do this, viral particles carrying a functional copy of the gene of interest and various nonviral gene delivery systems, including liposomes, nanoparticles, etc., can be used. In this review, we discuss the state of current knowledge regarding the molecular mechanisms and types of genetic mutations that lead to cystic fibrosis and highlight recent developments in gene therapy that can be leveraged to correct these mutations and to restore the physiological function of the carrier protein transporting sodium and chlorine ions in the airway epithelial cells. Restoration of carrier protein expression could lead to the normalization of ion and water transport across the membrane and induce a decrease in the viscosity of airway surface fluid, which is one of the pathological manifestations of this disease. This review also summarizes recently published preclinical and clinical data for various gene therapies to allow one to make some conclusions about future prospects for gene therapy in cystic fibrosis treatment.

## INTRODUCTION

Mucoviscidosis or cystic fibrosis (CF) is a rather common monogenic disease. CF is a congenital systemic disease caused by the mutated gene coding for the CF transmembrane conductance regulator protein (*CFTR*) [1]. The molecular pathogenetic mechanism of the disease is based on the dysfunction or total absence of the *CFTR*-encoded carrier protein that transports sodium and chlorine ions. This ion channel ensures normal functioning of epithelial cells in the lungs, intestines, pancreas, and some other organs.* CFTR *regulates sodium and chlorine ion transport across the membrane, as well as water exchange in secretory the epithelial cells in the respiratory, gastrointestinal, hepatobiliary, and reproductive systems [2, 3]. Impairment of the protein’s function causes a severe progressive pathology that clinically manifests itself in pulmonary (respiratory failure), pancreatic, and hepatic lesions (sometimes as severe as cirrhosis), as well as increased electrolyte content in the perspiratory secretion.

There are several forms of CF: 75–80% of cases are accounted for by a mixed pulmonary/intestinal form of CF; pulmonary CF is diagnosed in 15–20% of cases; and intestinal CF, in 5% of cases. Mixed CF is considered the most severe form of the disease, because it combines clinical signs of both the pulmonary and intestinal forms. In addition, one could argue for recognition of relatively rare forms, such as meconium ileus (15–20% of cases), anemic edematous CF, cirrhotic CF, and others. However, these classifications are mostly made for the sake of discussion, since a major respiratory tract lesion is often accompanied by digestive disorders. The same is true for the intestinal form of CF; i.e., intestinal lesions are often accompanied by bronchopulmonary lesions. The main complications associated with CF include pulmonary and gastric hemorrhages, intestinal ob- struction, bronchial hyperresponsiveness, edemas, abscesses, pneumo- and pyopneumothorax, pulmonary heart disease, maxillary sinusitis, liver cirrhosis, rectal prolapse, developmental impairments, sterility, diabetes mellitus, etc. [2].

According to the statistical data, about 650 newborns in Russia are diagnosed with CF every year [4], while the worldwide number is one diagnosis per 2,000–5,000 healthy newborns. The total number of CF cases in the United States and Europe is about 70,000 [5]. The disease affects males and females equally. Children are usually diagnosed with CF in their first years of life, because lesions to the affected organs (especially lungs and intestines) are clearly visible even at the early stages. Patients show multiple impairments in various systems, including the respiratory, digestive, locomotor, nervous, cardiovascular systems, etc. Exocrine pancreatic insufficiency (ductal dysfunction) is observed in 85–90% of cases. The average life expectancy for CF patients may be 30–40 years, with their quality of life directly depending on the scope of the specialized medical care they receive and the availability of symptomatic treatment. Despite that, up to 90% of CF patients die from pulmonary infections and associated complications [3].

Since CF is caused by a *CFTR *gene mutation, the disease is not fully reversible through the currently available methods. Until recently, CF management remained confined to symptomatic treatment; i.e., mucus thinning (mucolytics), bronchiectasis therapy, anti- inflammatory therapy, antibacterial therapy, and enzyme replacement therapy (in intestinal CF). All these therapies fail to increase the life expectancy of patients and only manage to temporarily improve their quality of life [6]. The development of *CFTR* modulator drugs (Vertex Pharmaceuticals) for pathogenetic therapy has significantly increased the life expectancy of CF patients, but the cause of the disease still could not be eliminated, and patients are condemned to expensive life-long therapy

On the other hand, the use of gene therapy aimed at restoring the function of the *CFTR *gene in epithelial cells offers new opportunities in the management of CF and other severe hereditary diseases, where gene therapy has already proved to be safe and efficacious. Rapid developments in genome editing technology leave us hopeful for the development of etiotropic therapy, making it possible to correct the *CFTR* mutation causing mucoviscidosis and, through that, improve the quality of life and life expectancy of CF patients.

## MOLECULAR-GENETIC MECHANISMS OF CF DEVELOPMENT

**Fig. 1:**
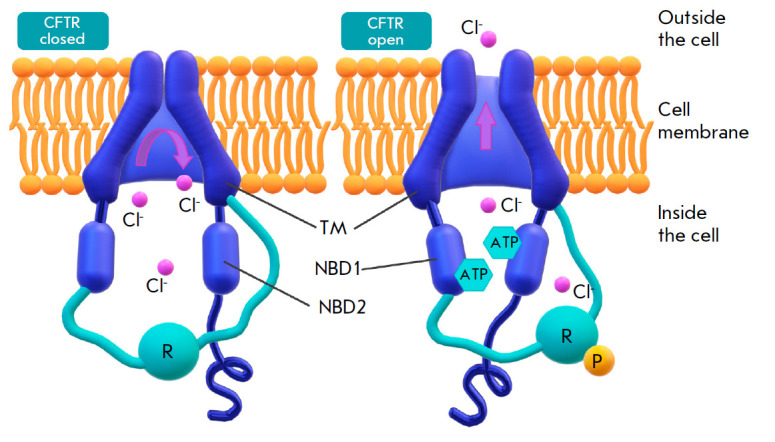
Schematic representation of a *CFTR *protein in the closed (left) and open (right) positions. TM – the transmembrane domains that form a channel for chloride ions transport. NSD1 and NSD2 – intracellular nucleotide-binding domains 1 and 2. R – the regulatory domain that contains phosphorylation sites (P). Channel activation requires the presence of a phosphoric acid residue on the regulatory domain. NSD1 and NSD2 bind and hydrolyze ATP, resulting in the opening of the channel through interaction with transmembrane domains [7]

*CFTR *is a transmembrane protein localized on the apical surface of epithelial cells. ATP binding of this protein changes its conformation inside the channel protein ensuring extracellular transport of Cl− ions. In turn, the termination of ATP hydrolysis leads to channel closing (*Fig. 1*).

It is known that maintaining normal osmotic pressure and fluid circulation in the intercellular space requires the presence of sodium and chlorine ions near the outer membrane. In addition, a controlled continuous flux of chlorine ions across the membrane is a necessary condition for proper functioning of epithelial cells in the lungs, intestines, sweat glands, and other organs. Impairment of the transmembrane transport of chlorine ions changes transmembrane conductance for water molecules and, as a result, causes dehydration and increased viscosity of the secretion. This is what determines the organs primarily affected by CF: a thick viscous secretion is formed on the epithelial surface and blocks bronchopulmonary airways and glandular lumens, which interferes with the normal functioning of the respective organs [2].

**Fig. 2:**
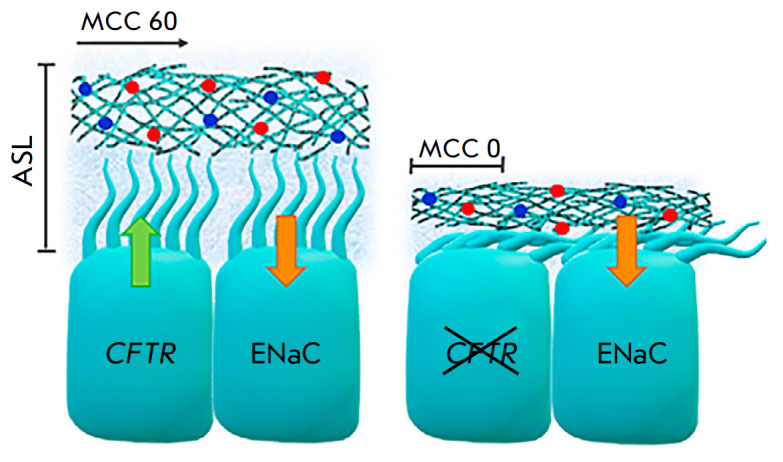
In healthy individuals (left), the thickness of the airways mucosal layer (ASL, airway surface liquid) is a result of the normal functioning of the *CFTR *and ENaC channels. MCC – mucociliary clearance that is the airway clearance rate due to mucus movement (in μm/sec). In cystic fibrosis (right), due to a defective *CFTR*, a decrease in the number of chloride ions leads to excessive transport of sodium ions resulting in dehydration of the airway epithelium surface, increased secretion viscosity, and compression of the cilia. Weak secretion mobility triggers an inflammatory reaction, and it is also an ideal environment for the reproduction of pathogenic microorganisms [9, 10]

Secretion and absorption are two opposite processes associated with the transport of the electrolytes regulating the viscoelastic properties of the liquid component of exocrine secretions. According to available data, electrolyte transport dysfunctions in CF occur both at the level of salt absorption and at the level of fluid absorption and secretion, which are mediated by anions [8]. A decrease in chlorine ion content in the intercellular space activates the epithelial sodium channel (ENaC), which increases the Na content in the cell (*Fig. 2*). It, in turn, boosts the absorption of Cl– ions and water and causes abnormalities in transepithelial electrical potential difference. As a result, the volume of fluid on the airway surface decreases, its viscosity increases significantly, and the clearance rate on the ciliated epithelial surface is sharply reduced (*Fig. 2*). Such processes in the lungs lead to dehydration of the airways and, subsequently, a reduction in the cleansing effect of epithelial cilia and mucosa in general. What is more, mucus congestion also favors the rapid development of infections [9].

The produced secretion is a polymeric mesh consisting of O-glycosylated glycoproteins (mucins) secreted as threads, forming a porous structure [11, 12]. The viscoelastic properties of the secretion and its structure under normal physiological conditions are specifically adapted to trap and remove inhaled particles and bacteria. Increased secretion viscosity in CF causes mucin plaques and a reduction in pore size from 0.2–1 μm to under 0.1 μm. As a result, neutrophils acting as the first line of immune defense against bacteria are unable to migrate through the mucus. At the same time, the bacterial macrocolonies formed on the thick mucus are especially resistant to the immune response and antibiotics, which further complicates the therapy [13]. Chronic infections caused by unrestricted proliferation of bacteria on the airway surface are considered the main cause of death in CF [14].

## CFTR GENE MUTATIONS

**Fig. 3:**
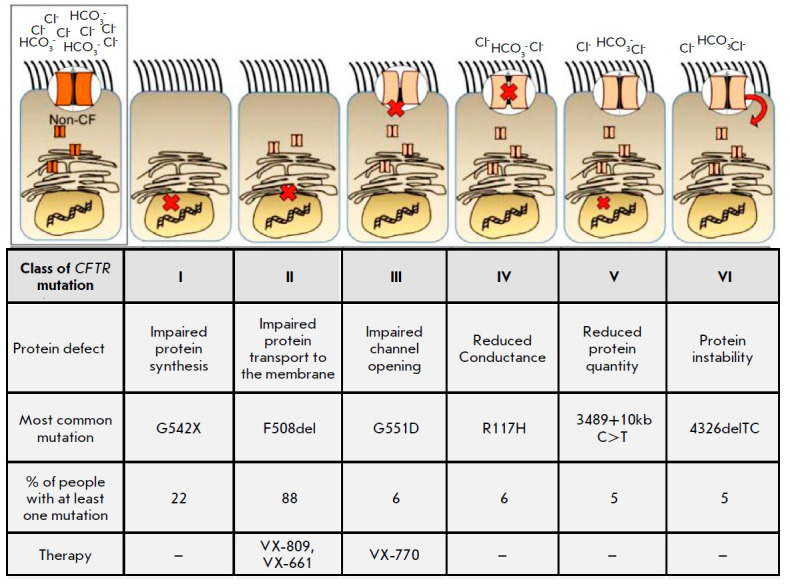
Types of *CFTR *mutations and therapies approved by the FDA for the treatment of the conditions associated with these mutations. CF patients may have more than one mutation. *Fig.* adapted from [23]

CF is an autosomal recessive disease caused by mutations in the *CFTR *gene identified in 1989 by a research team headed by Lap-Chee Tsui [15, 16]. The* CFTR *gene is localized on chromosome 7 and consists of 27 exons and codes for a protein composed of 1,480 amino acid residues. Over 2,000 mutations in the* CFTR *gene have currently been described, and the list is updated on a regular basis, but only 250–300 of these mutations have pathological consequences, and among those only 20 are relatively common (over 0.1% of patients) [17]. Five classes of mutations (seven, according to some authors) are identified based on the associated defects (*Fig. 3*). Class I–III (severe) mutations are associated with a fundamental *CFTR *dysfunction; and class IV–V (mild) mutations, with the residual function of the *CFTR *protein [18]. Various mutations in the *CFTR *gene may impair the synthesis, processing, stability, and functioning of the *CFTR* protein, as well as its intracellular transport from the endoplasmic reticulum to the Golgi complex and degradation, which leads to a variety of phenotypic manifestations [19].

**Class I mutations**

Class I mutations (G542X, W1282X, R553X, 2143delT, 1677delTA) are observed in about 10% of CF patients. If the gene includes this type of mutation, then the *CFTR *protein is not synthesized at all or its shortened variant is synthesized and degraded. This class of mutations includes nonsense mutations, frameshift mutations, and splicing site mutations causing the generation of a stop codon, premature termination of protein synthesis, and production of an enzyme that can no longer function as the initially synthesized protein [19].

**Class II mutations**

Class II missense mutations (del F508, del I 507, N1303 K, S541 I, S549 R) are considered the most common in CF patients. Among those, F508del, i.e., deletion of phenylalanine residue in position 508, occurs most often. About 70% of patients have mutations in both copies of the *CFTR *gene (homozygous), and 90% of patients have at least one mutant allele [20]. The most severe course of the disease affects homozygous patients, while heterozygous *CFTR*-F508del with one healthy copy of the gene show no signs of the disease.

The F508del mutation causes errors in protein folding and its further processing, which is why most mutant molecules are unable to reach the cellular membrane and are destroyed. It should be noted that about 1% of these molecules still manage to reach the cellular surface, but since the mutation impairs the mobility of the domains associated with opening and closing of the channel, protein effectiveness remains very low [21]. On top of that, the protein is removed from the surface and destroyed in a matter of several minutes [22].

**Class III mutations**

Class III missense mutations (G551 D, G1224 E, S1255 P) affecting the regulation of ion channel opening are observed in about 4–5% of CF patients. Proteins with this mutation reach the apical membrane, but conductance and permeability of the channel are impaired. Here, the conversion of the glycine residue in position 551 of NBD1 into aspartic acid (G551D) is the most common mutation. This mutation leaves the channel closed most of the time [19, 21].

**Class IV mutations**

Class IV mutations are the rarest ones (about 1.7%). These mutations (R117H, R334W, R347P) reduce chlorine ion transport through the open *CFTR *channel [9] and convert positively charged arginine residues in the *CFTR *channel into noncharged residues (presumably, the presence of positive charges in the channel is required for Cl− ion transport). These mutations in CF patients are usually associated with a mild course of the disease, often without pulmonary or pancreatic signs.

**Class V–VI mutations**

In some cases, clinicians also identify class V–VI mutations, where the functional *CFTR *protein is produced, but its synthesis is inhibited and it is quickly removed from the cellular surface, which leads to insufficient content of the protein. These mutations are associated with a relatively mild course of the disease [17].

## GENE THERAPY OF CF

The discovery of *CFTR *modulators that can correct the functioning of the defective protein has had a positive effect on life expectancy and quality of life and given hope to many CF patients. However, about 10% of patients are unresponsive to *CFTR *modulators because *CFTR *is not synthesized at all or is only synthesized in low quantities. In addition, clinical trials (CT) show that 10–20% of CF patients have individual intolerance to modulator drugs [24].

This taken into account, new approaches to CF management are being developed, including the ones using gene therapy methods to deliver nucleic acids to the affected cells to address the primary (genetic) cause of the pathology and, through that, mitigate the course of the disease. Even though multiple organs are affected by the CF, lungs are the main target of the gene therapy, since 90–95% of deaths from the disease are due to severe pulmonary lesions. The key strategy in CF gene therapy is to ensure that the* CFTR *gene is delivered to the airway epithelial cells. Here, the delivery method should be selected taking into account the significantly reduced efficacy of aerosol administration due to the thick secretion in the bronchioles. The latter also imposes additional restrictions on the gene therapy, since the vector should not only ensure the effective expression of the functional* CFTR *protein but should also penetrate submucosal glandular cells and the superficial mucosal epithelium covered by the thick secretion [2].

CTs of gene therapy drugs, where the genes of interest are delivered to nasal and bronchial airway epithelium in CF patients using both viral and nonviral systems, have been taking place since 1993. So far, over 27 CTs of gene therapy in CF involving over 600 patients have been completed but none of them has shown significant success for one reason or another (*Table 1*).

**Table 1:** Selected CTs of CF gene therapy^*^

CFTR delivery method	Administration method	Clinical trials	Reference
Adenovirus (Ad)	Nasal administration, endobronchial administration	NCT00004779 NCT00004287	[26, 27, 28, 29]
Adeno-associated virus (AAV)	Maxillary gland administration, nasal administration, endobronchial administration	NCT00073463 NCT00004533	[30, 31, 32]
Lentivirus (LV)	Intranasal administration (perfusion)	Preparation stage	[33]
Nanoparticles (liposomes), synthetic polymers	Aerosol administration (nebulizer), intranasal administration	NCT01621867 NCT00789867 NCT00004471 NCT00004806	[34, 35, 36, 37, 38]
Single-stranded antisense RNA-oligonucleotide (QR-010)	Intranasal administration	NCT02564354 NCT02532764	[39]

^*^All CTs are completed.

It should be noted that continuous renewal of airway epithelium necessitates repeated delivery of the gene of interest, which restricts the use of viral vector systems, because the repeated administration often triggers an immune response resulting in vector elimination. In addition, the lack of adequate *in vivo *models for testing the efficacy of new vectors also hinders the progress in the research. Therefore, despite the initial enthusiasm, there is still no FDAapproved gene therapy for CF [25]. Nevertheless, advances in vector development, better understanding of various vector serotypes, and development of new* in vivo *CF models has sustained the search for more effective CF gene therapy [5].

**Gene delivery using adenoviral (Ad) vectors**

The first CTs of CF gene therapy were aimed at using Ad to deliver a healthy copy of a gene into airway epithelial cells (*Table 1*). Two CTs using first-generation Ad have been completed [26, 27, 28, 40, 41]. But despite the efficacy of the approach in cell models and* in vivo*, the CT results raised the issue of the questionable safety of the vectors for humans. Congenital and cellular immunity hindered the long-term effect of Ad-based vectors: observations showed increased alveolar inflammation, accompanied by an increase in serotype-specific neutralizing antibodies, which rendered the repeated administration of viral particles ineffective [23].

In later designs, the gene was delivered using an improved Ad platform in the form of a helper-dependent adenovirus (HD-Ad) devoid of viral genes, which made it possible to neuter the T cell response to the viral protein that was a feature of the firstgeneration Ad vectors. Nevertheless, the adaptive immune response of CD8^+^ T cells with HD-Ad epitope presentation by dendritic cells remained present [42].

HD-Ad was used in the lungs in combination with lysophosphatidylcholine (LPC) with the intention to destroy the thick secretion layer and ensure better access to the basolateral cell surface for infection. This strategy resulted in lengthier gene expression *in vivo* compared to the first-generation Ad and demonstrated effective gene delivery to the airways in mice, pigs, and ferrets [43, 44].

A possible modification of the Ad platform is to use piggyBac transposons with their cut-and-paste mechanism for gene transfer*. *Transposase-mediated piggyBac insertion in the recombinant Ad produced a hybrid vector piggyBac*/*Ad, which made it possible to effectively express the gene of interest in the lungs of pigs [45].

Another approach to CF therapy, which is yet to be studied in detail, is the use of genome editing tools TALEN (Transcription Activator-Like Effector Nucleases) and CRISPR (Clustered Regulatory Interspaced Short Palindromic Repeats)/Cas9. These relatively recent molecular methods of genome editing have already proved their efficacy and reliability [46]. The relative safety and significant capsid size of HD-Ad vectors (36 kbp) make it possible to transfer several constructs at the same time, which allows for the use of site-specific nucleases for targeted insertion of a delivered gene at a desired locus. This specific insertion of a healthy gene copy is advantageous compared to the correction of the mutated protein, because here CF therapy no longer depends on the* CFTR *mutation type. An example of this approach is presented in Xia et al. [47], where an expression cassette with the *CFTR *gene was inserted at the *AAVS1* locus *in vitro *using a HD-Ad vector simultaneously carrying the TALEN nuclease. Expression of *CFTR* mRNA and restored protein function were observed in the cells transduced by the vector with this expression cassette [47]. A similar approach with an HD-Ad vector for precise delivery of CRISPR/Cas9 and a DNA copy at the GGTA1 locus in the genome of airway epithelial cells was used *in vitro *and *in vivo *in pigs. It transpired that the transduced cells expressed functional *CFTR *at mRNA and at the protein levels both in *in vitro *and in *in vivo *models. An engineered cell line CFTR-/- of pig epithelium was developed for *CFTR *protein expression assessment after transduction with CRISPR/Cas9. Measurement of *CFTR *channel activity in the transduced *CFTR*-/- cells showed restoration of the anion transport function [48, 49]. These data allow us to anticipate a new nuclease-based approach to CF gene therapy in the near future.

**Gene delivery using adeno-associated viral (AAV) vectors**

Replacement of a mutated CFTR protein gene with its functional copy turned out to be a rather complex undertaking, and following the failure with first-generation Ad vectors the search for alternative approaches in gene delivery to target cells was initiated. The reports from the CTs using the AAV2 vector (*Table 1*) showed that introduction of the vector into the lungs of CF patients did not cause significant side-effects, but the efficacy was disappointing, since none of the CTs demonstrated significant *CFTR* expression or correction of pathological CF manifestations. The lack of success could be explained by the insufficient efficacy of gene insertion (possibly due to the inability of viral particles to penetrate the thick secretion layer in the airways), insufficient promoter strength in the expression cassette, or immune response of the host to the introduction of the viral vector [50]. Hence the recent efforts to improve the tropism of AAV vectors, identify new serotypes, new promoters, new methods to enhance the expression of the target protein and its persistence in the lungs, as well as new approaches to immunogenicity reduction. At the same time, new *in vivo *models, including pigs [51], sheep [52], ferrets [53], and mice [54], were being developed, which, along with the conventional *in vitro* tests in human epithelial cells, would make it possible to carry out more effective preclinical trials for the CF gene therapy.

For example, the AAV virus with high airway epithelial tropism was selected based on *in vivo *experiments in pigs [51]. Improved AAV2H22 capsid based on AAV2 with five-point mutations made specific infection of airway epithelium in pigs 240 times as effective. One of the key parameters indicating phenotypic efficacy of the therapy is Cl^-^ transport. Introduction of AAV2H22-CFTR into the airways of* CFTR*-null pigs lacking a functional *CFTR *gene resulted in *CFTR *expression in epithelial cells, restoration of anion transport, and normalization of the pH of the secretion on the airway surface and its bactericide properties [51].

Gene expression efficacy was also increased using the AAV vector including the CFTRDR gene of the shortened protein driven by a short cytomegalovirus promoter CMV173. Transduction of organoids by AAV-CFTRDR resulted in restored *CFTR *function. In addition, changes in the potential difference on the epithelial cell membrane in nasal airways were recorded, which was an indication of the restoration of the normal phenotype in mice carrying the most common CF mutation, ΔF508 [54]. The problem of the limited size of the genetic construct packed in AAV2 may be solved by developing a short synthetic promoter [55] or obtaining a *CFTR *gene with partial deletion of the regulatory domain [56].

In addition, a new chimeric vector, AAV2/HBoV1, obtained by pseudotyping the AAV2 genome into a capsid of human bocavirus, HBoV1, infecting human airways and characterized by high tropism for the apical surface of airway epithelial cells in humans was tested [57]. The capsid size was increased as a result, which made it possible to use a stronger promoter and a complete *CFTR *gene [58]. The ability of rAAV2/ HBoV1 to transduce pulmonary epithelial cells in ferrets (*Mustela putorius furo*) made it possible to create* in vivo *models for preclinical trials [53].

Testing of nine characterized AAV vector serotypes in the epithelial cells and lungs of mice resulted in identification of the AAV6 vector with the highest tropism for pulmonary epithelial cells in mice and humans [59, 60]. It transpired that the transduction efficiency of AAV6 in the airway epithelial cells of mice reached 80% and that its immunogenicity was lower than that of the AAV2 vectors, which makes AAV6 a preferable vector for gene therapy of CF and other pulmonary diseases [61]. To further boost the transduction efficiency of the AAV6 vector in epithelial cells, a point mutation was introduced into the gene coding for an atypical amino acid residue, F129, usually present in the capsid protein. The resulting AAV6.2 vector showed higher transduction efficiency in both the airway cells of mice and HAEC (human airway epithelial cells) cultures. Stable expression of the transgene intranasally administered (2 × 10^11^ viral particles) to macaques for 72 days was observed [59]. The advantage of the AAV6 vector in penetrating mucus obtained from CF patients was also shown in the new mouse model most accurately mimicking the pulmonary pathophysiology in obstructive pulmonary diseases. The point mutation in the capsid protein seems to point to the potential mechanism used to avoid AAV6 adhesion to the polymeric mesh representing the mucus in CF and prompting the attack against other AAV vector serotypes [62].

It should be noted that only a few pharmaceutical companies are currently involved in the development of AAV-based CF gene therapy. According to Abeona Therapeutics [63], preclinical trials of ABO401, a new-generation capsid AAV204 developed by the company and carrying a functional copy of the human mini-*CFTR *gene, show that the product effectively restores the main phenotypic attribute of CF, i.e., chlorine channel functioning, in *in vitro *and *in vivo *models. In addition, AAV204 more specifically targets pulmonary cells and also transduces bronchial and nasal epithelial cells in CF patients (*CFTR* expression rate 3–5 times higher compared to the AAV6 vector).

In addition, Spiro-2101 by Spirovant Sciences, designed for CF therapy was certified by the FDA as an orphan drug in 2020, which allowed the company to accelerate its clinical trials and take the drug to the market. Spiro-2101 also includes a new AAV capsid with improved tropism for airway epithelial cells for the delivery of a functional copy of the *CFTR* gene.

**Gene delivery using lentiviral vectors**

Lentivirus-based vectors are widely used in gene therapy as well. Their beneficial aspects include low immunogenicity, ability to infect various cell types and integrate consistently into the genome to ensure long-term expression and preservation of the gene in cell division. Nevertheless, it should be mentioned that consistent integration into the genome may lead to insertional mutagenesis and, as a result, a risk of tumor transformation (oncogenesis) [64]. All existing approaches to CF therapy using lentiviral vectors (LV) are currently undergoing preclinical trials, but recent advances in the application of improved lentiviral vectors in various CTs have shown that they are safe to use in CF therapy [65].

Studies into the primary epithelial cultures of CF patients and animal models have shown the long-term phenotype correction and low immunogenicity carried by lentiviral vectors. In particular, the restoration of *CFTR *channel functioning in the airways of pigs after transduction with the feline immunodeficiency virus (FIV) pseudotyped with the GP64 protein to ensure apical tropism for HAE-ALI (human airway epithelium cultured on an air-liquid interface) cells was demonstrated in *in vivo *experiments. A significant increase in Cl^-^transepithelial transport and normalization of the pH of the tracheal surface fluid and its bactericide properties were observed two weeks after FIV-CFTR aerosol administration into the nose and lungs [66].

Another experimental design involved the simian immunodeficiency virus (SIV) pseudotyped with the Sendai virus fusion protein (F), hemagglutinin, and neuraminidase (HN). Preclinical trials showed that* CFTR *gene transfer into the lungs using this vector ensured more efficient transduction of human bronchial epithelial cells and the pulmonary epithelium of mice *in vivo *compared to nonviral transfer and did not trigger any immune response [33].

In 2017, Alton et al. analyzed the results of several preclinical trials to select the most promising vector type for initiation and planning of the first-in-man CT using lentiviral transfer of the *CFTR *gene. A lentivirus vector rSIV.F/HN ensuring the expression of functional *CFTR *with efficacy of 90–100% in clinically relevant delivery devices was considered the lead candidate. These data support the idea of using this vector in the first CT in CF patients [33]. Yet the CT has not been initiated, probably a clue that the vector requires additional preclinical trials and proof of efficiency as a CF gene therapy.

**Non-viral gene delivery using liposomes and polymeric nanoparticles**

The benefits of liposomal gene transfer include simplicity in scaling up the final formulation of the product and a size suitable for large DNA molecules. In 2015, one of the largest CTs, where pGM169/GL67A liposomes were used for *CFTR *delivery, showed that the product was safe in CF [67]. Safety with repeated administrations of the product was confirmed in a later CT using pGM169/GL67A liposomes. It was shown for the first time that gene therapy is capable of slowing down the deterioration of the pulmonary function in CF patients but that the relief was still insufficient for researchers to recognize the therapy as efficient [34].

**Fig. 4:**
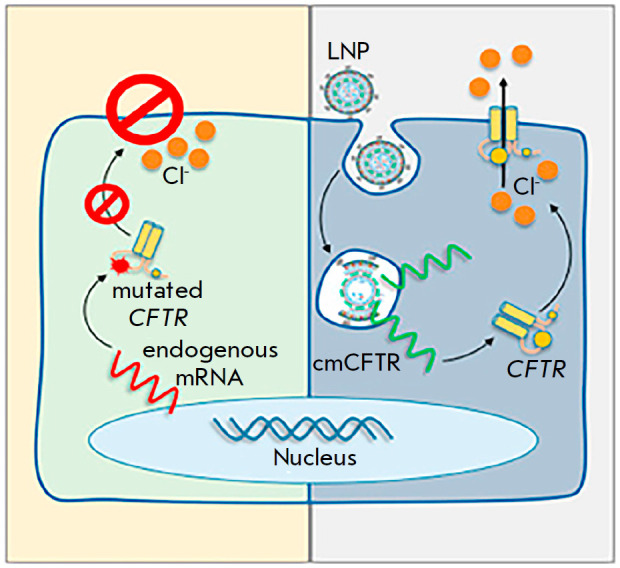
LNP-cmCFTR delivery. *Fig*. adapted from [68]

In recent years, research efforts have been directed toward increasing efficiency in liposome-based gene delivery (*Fig. 4*). In particular, it was discovered that the use of clinically relevant liposomal nanoparticles (LNP) for the packaging and delivery of chemically modified *CFTR *(cmCFTR) mRNA into the bronchial epithelial cells of CF patients increased the quantity of the *CFTR *localized on the membrane and restored the function of chlorine channels [68].

In addition, intranasal administration of LNP-cm*CFTR *resulted in restored Cl^-^transport in the airway epithelium of CFTR-KO mice for 14 days. *CFTR *functional activity reached its peak on the 3^rd^ day after transfection, which was supported by a restoration of Cl^-^flux to 55% of that in healthy mice. These results are comparable in efficiency with Ivacaftor (*CFTR *modulator) and support the idea of using LNP-cmCFTR to correct for CF and other monogenic diseases [68].

There are also a number of polymer-based methods, including dense polyethylene glycol (PEG) coating of particles to ensure that they penetrate the thick mucus layer *in vitro *and, thus, increase transfection efficiency in the lungs of mice *in vivo *[69]. Also of interest is the use of biodegradable triplexforming peptide nucleic acids (PNA) binding to genomic DNA and forming PNA/DNA/PNA triplexes that can stimulate the restoration of endogenous DNA. Delivery of these complexes, along with the corrective gene, results in site-specific gene correction [70]. In this case, introduction of the donor DNA* in vivo *into nasal sinuses and the lungs of homozygous ΔF508del mice caused significant mutation correction in airway epithelium and mitigated the course of the disease [71].

In addition, the first attempt at systemic introduction of the improved polymeric nanoparticles PNA LNP carrying DNA-editing agents and characterized by higher cell permeability and efficiency of mutation correction was described. I/V administration of these particles led to a more adequate biodistribution, with particles accumulating in the airways and gastrointestinal tracts of mice, and *CFTR *functions in epithelial cells fully restored. This was the first successful case of systemic introduction of nanoparticles as CF gene therapy [72].

**Antisense oligonucleotides**

It is known that oligonucleotides and their complexes have been used as therapeutic molecules for the restoration of DNA modifications (DNA repair) [73]. These oligomers, including RNA- and/or DNAnucleotides, are used for site-specific repair of defective DNA.

Recently, ProQR Therapeutics have completed two CTs looking into the possibility of RNA-mediated* CFTR *gene correction. Intranasal administration of single-stranded antisense RNA (eluforsen, QR-010) designed for specific binding to the F508del domain in mRNA and the restoration of *CFTR *function in airway epithelium was used in the CTs. Preliminary *in vitro *and *in vivo *studies in mice showed that QR-010 was able to quickly diffuse through the CF-like secretion, presumably due to its small size and negative charge. QR-010 remained stable even when combined with conventional CF therapies and under bacterial infection. On top of that, positive changes in chloride transport were observed [74, 75, 76, 77]. The CT results showed that QR-010 restored the *CFTR *function in homozygous *CFTR*-F508del patients: A clinically significant improvement in the *CFTR *function, demonstrated by stabilization of the Cl and Na transport parameters, was observed after three intranasal doses for 4 weeks Cl and Na [78].

## CONCLUSIONS

Using preclinical models and clinical trials in CF, it has been shown that some advances have already been made in the use of gene therapy methods for the delivery of functional *CFTR *gene copies. Nevertheless, the problem of inefficient *CFTR* gene delivery to bronchopulmonary airway epithelial cells still stands. No optimal approach has yet been found to ensure protein expression in epithelial cells in the quantities required for a pronounced therapeutic effect. It should be taken into account that viral delivery of genetic material may naturally trigger an immune response to the viral capsid upon repeated administration and, thus, a reduced therapeutic effect, while non-viral carriers possess enough permeability to penetrate a thick mucus layer. Despite the fact that there currently are no FDA-approved CF gene therapies, the critical factors that hobble therapeutic efficiency have already been identified and efforts have been initiated to overcome them. Based on the data available, more efficient delivery methods will appear, efficiency in the penetration through the thick secretion layer will increase, and the immune response to therapy will be minimized. The rapid developments in gene engineering technology of recent years provide hope that etiotropic CF therapy will become a reality in the near future.

## Abbreviations

CFTR: cystic fibrosis transmembrane conductance regulator
NBD: nucleotide-binding domain
TM: transmembrane
MCC: mucociliary clearance
CT: clinical trials
Ad: Adenovirus
AAV: Adeno-Associated Virus
LV: Lentivirus
LNP: Liposomal NanoParticles
FDA: Food and Drug Administration.
